# Transplant Tolerance Induction in Newborn Infants: Mechanisms, Advantages, and Potential Strategies

**DOI:** 10.3389/fimmu.2016.00116

**Published:** 2016-04-07

**Authors:** Hua Pan, Aram Gazarian, Jean-Michel Dubernard, Alexandre Belot, Marie-Cécile Michallet, Mauricette Michallet

**Affiliations:** ^1^Chair of Transplantation, VetAgro Sup-Campus Vétérinaire de Lyon, Marcy l’Etoile, France; ^2^Plastic and Reconstructive Surgery Department, Xijing Hospital, The Fourth Military Medical University, Xi’an, China; ^3^Department of Hand Surgery, Clinique du Parc, Lyon, France; ^4^Department of Transplantation, Hôpital Edouard Herriot, Lyon, France; ^5^International Center for Infectiology Research (CIRI), Université de Lyon, Lyon, France; ^6^Cancer Research Center Lyon (CRCL), UMR INSERM 1052 CNRS 5286, Centre Leon Berard, Lyon, France; ^7^Department of Hematology, Centre Hospitalier Lyon-Sud, Pierre Benite, France

**Keywords:** transplantation, tolerance induction, infants, chimerism, stem cells

## Abstract

Although several tolerance induction protocols have been successfully implemented in adult renal transplantation, no tolerance induction approach has, as yet, been defined for solid organ transplantations in young infants. Pediatric transplant recipients have a pressing demand for the elaboration of tolerance induction regimens. Indeed, since they display a longer survival time, they are exposed to a higher level of risks linked to long-term immunosuppression (IS) and to chronic rejection. Interestingly, central tolerance induction may be of great interest in newborns, because of their immunological immaturity and the important role of the thymus at this early stage in life. The present review aims to clarify mechanisms and strategies of tolerance induction in these immunologically premature recipients. We first introduce the discovery and mechanisms of neonatal tolerance in murine experimental models and subsequently analyze tolerance induction in human newborn infants. Hematopoietic mixed chimerism in neonates is also discussed based on *in utero* hematopoietic stem cell (HSC) transplant studies. Then, we review the recent advances in tolerance induction approaches in adults, including the infusion of HSCs associated with less toxic conditioning regimens, regulatory T cells/facilitating cells/mesenchymal stem cells transplantation, costimulatory blockade, and thymus manipulation. Finally, IS withdrawal in pediatric solid organ transplant is discussed. In conclusion, the establishment of transplant tolerance induction in infants is promising and deserves further investigations. Future studies could focus on the selection of patients, on less toxic conditioning regimens, and on biomarkers for IS minimization or withdrawal.

## Introduction

The neonatal stage is an immune “window phase” susceptible to the induction of transplantation tolerance, as described in animal models. The phenomenon of fetal immunological tolerance and chimerism was noticed in 1945 by Owen (1), who discovered that dizygotic twin cattle had two distinct sets of red blood cells (RBCs). Indeed, each twin had its own set of RBCs, as well as another set, that could only have been derived from the other twin sibling during fetal life. In 1949, Burnet introduced the concept of “self” and “non-self” in immunology (2). He hypothesized that, if cells from a genetically distinct individual are implanted and established in the embryo, no antibodies should develop against the foreign antigen later in life. This hypothesis was proven in 1953 by Medawar and colleagues in a murine experimental model, in which allogeneic splenocyte injections during the fetal or neonatal stage resulted in chimerism and neonatal tolerance without any conditioning regimen under certain circumstances (3). Such neonatal tolerance induction protocols, though efficient in rodents, cannot be transposed into large animals and human neonates who are born with an almost entirely functional immune T cell compartment (4, 5) (Table 1). Full tolerance of allografts without immunosuppressive regimens has rarely been observed, although such cases are described in pediatric patients with severe combined immunodeficiency (SCID) that received stem cell transplantation (SCT) (6). In adult human recipients, non-myeloablative transplants and immunosuppressive regimens followed by the infusion of donor-derived hematopoietic progenitor cells have been proven to be efficient in inducing tolerance to HLA-mismatched renal allografts (7). Nevertheless, such non-myeloablative tolerance induction protocols have not been used in young pediatric solid organ transplant (SOT) recipients. Indeed, a major concern remains with respect to the toxicity of non-myeloablative conditioning regimens and the effectiveness of present tolerance induction protocols.

**Table 1:** **Comparison of immune maturation between mouse and human (5)**.

Functions	Mouse	Human
Length of gestation	21 days	40 weeks
Lymphohematopoietic cells colonize primordial thymus	Days 11–12	Week 9
Morphologic division of thymus into cortex and medulla	Days 13–14	Weeks 11–14
Expression of γδ-TCR and αβ-TCR	Days 14–16	Weeks 11–13
Proliferative response demonstrable in MLR	Days 16–18	Week 12 (thymus), week 19 (spleen) (weak until week 23)
Mitogen responsiveness	Day 18 (thymus) (to some mitogen only after birth)	Weeks 13–14 (thymus), weeks 16–18 (spleen, peripheral blood)
Cytotoxic response demonstrable in (CML)	Weak until postnatal day 7	Beginning from about weeks 20–23 (thymus)

*TCR, T-cell receptor; MLR, mixed lymphocyte reaction; CML, cell-mediated lympholysis*.

At the present time, SOT is more and more frequently performed in infants or even neonates. Approximately 25% of the 450–550 pediatric heart transplants reported to the Registry of the International Society for Heart and Lung Transplantation every year concern young infants (<1 year) (8). Compared to adult patients, the immune system of these young patients still demonstrates immature features and susceptibility to transplant tolerance. Clearly, spontaneous engraftment without immunosuppression (IS) has been observed in 25–38% of pediatric recipients of liver transplants (9). Besides, ABO-incompatible (ABOi) SOT and B cell tolerance can be achieved in young infants, due to the relative immaturity of T cell-independent antibody responses (5). Under such circumstances, there is a strong interest in understanding and implementing tolerance induction in pediatric transplantation. The present study reviews the current knowledge and recent progress in the field of neonatal tolerance and pediatric transplant tolerance, in order to evaluate the advantages and challenges associated with the establishment of tolerance induction protocols in infants and neonates.

## Mechanisms of Neonatal Tolerance in Mice

Although neonatal tolerance was discovered 60 years ago, the underlying mechanisms remain to be thoroughly investigated. As demonstrated by Medawar and colleagues in their publication “‘Actively Acquired Tolerance’ of Foreign Cells” (3), neonatal tolerance is based on two main principles, namely, (i) acceptance of foreign cells from the original inoculum. Indeed, the engraftment of allogeneic splenocytes or bone marrow cells (BMCs) in neonatal recipients does not require any conditioning regimen prior to transplantation. (ii) Tolerance to transplanted skin grafts from the same donors in adult recipients. The latter should be regarded as the maintenance of neonatal tolerance in adulthood. It is widely accepted that the tolerance to skin graft in adult life relies on central tolerance mechanisms that lead to the clonal deletion of donor-reactive thymocytes from the T cell repertoire, which arises from the contact and interaction between recipient thymocytes and thymic antigen-presenting cells (APCs), derived from recipient and donor precursor cells (4). Tolerance induction through donor-derived stem cell infusions in the case of SOT is at least partially based on central tolerance mechanisms. Regarding the tolerance of foreign cells in the original inoculum, the explanation is more obscure and is still a matter of debate. Several hypotheses and models have been proposed, including the active and passive models based on the “self/non-self” notion first voiced by Burnet. The T helper 1 (Th1) to Th2 immune deviation in the neonatal stage and the Th17 immune subpopulation were also proposed to be important factors facilitating tolerance to the original inoculum. Finally, the “danger” concept proposed by Matzinger in the immunology field in 1990s (10) may also provide some useful clues as to these underlying mechanisms.

### The Passive Model

The passive model is based on the clonal selection theory (2). It is a thymus-mediated central tolerance model, suggesting that neonatal mice present an immature immune system, and are, therefore, incompetent to mount a cytotoxic T lymphocyte (CTL) response to reject the donor cells. This would lead to the engraftment of donor cells in the neonatal mouse recipients and to their circulation to the thymus to induce tolerance by clonal deletion, such as natural self-tolerance. Natural self-tolerance is shaped by self-antigens expressed and presented by various types of thymic APCs, such as medullary thymic epithelial cells (mTECs) and thymic dendritic cells (DCs), present in the medulla (11). Depending on the quality and/or quantity of the overall signal delivered during the interactions between thymocytes and APCs, self-reactive thymocytes undergo either apoptosis (clonal deletion, e.g., negative selection), anergy, or the generation of central regulatory T cells (Tregs).

According to the passive model, when semiallogeneic splenocytes are infused intravenously to induce neonatal tolerance, donor antigen-specific thymocytes should be quickly depleted in the thymus during the process of negative selection. This was confirmed experimentally, since within 72 h of injection, thymocytes that were able to respond to alloantigens from the tolerance-conferring inoculum were no longer detected in the mixed lymphocyte reaction (MLR) (12, 13). Moreover, recipient T cells with receptors for donor-derived molecules could no longer be detected. During the same interval, cells bearing donor alloantigens entered into the thymus, spleen, and bone marrow of the host, creating a chimerism status in the recipients (12, 13).

The importance of chimerism for the induction and maintenance of central tolerance within the thymus was also investigated in the passive model. In the study by Hosono et al., recipient mice were neonatally tolerized by the intravenous administration of either BMCs or peritoneal cavity (PerC) cells from F1 donors. BMCs inoculation resulted in 1-year-long deletion of donor-reactive T cells, which was significantly different to the transient loss (1 week after birth) of donor-reactive T cells observed after PerC cell inoculation. The tolerant state correlated well with the degree and persistence of the intrathymic presence of F1 type cells (14). Hosono and colleagues next revealed that inoculated cells accumulating in the thymic medulla, but not in the cortex, induced tolerance to the MHC-II alloantigens (15). Eto et al. studied the role of chimerism in the establishment of allograft tolerance in adult mice conditioned with cyclophosphamide and then receiving infusions of allogeneic BM and spleen cells. Even when cells were infused at a concentration of 1.2 × 10^8^ (10 times more than in neonatal mice), neither mixed chimerism nor clonal deletion of antigen-specific thymocytes was observed in the thymus of 2-week-old mice, whereas both were observed at 5 weeks and lasted for more than 10 weeks following the treatment. These results suggest that micro-anatomical barriers that limit the entry of foreign cells are less stringent in the neonatal rather than in the adult thymus. This difference may explain why transplantation tolerance is easier to achieve in murine neonates (16).

### The Active Model

The active model is based on the immune network theory. It suggests that during the neonatal stage, the introduction of (CBA × A) F1 antigen into the developing immune system of recipient CBA mice may favor a “network interaction.” Hence, the F1 antigen recognized by nascent receptors expressed by specific peripheral T cells should be especially immunogenic to their “anti-idiotype” counterparts (suppressor T cells) and should selectively facilitate the expansion of this population in order to predominantly suppress the alloreactive response (17). The existence of “suppressor T cells” as proposed by the active model was then denied by a better understanding of the T cell receptor (TCR), but this notion initiated the discovery of Treg populations. However, the exact role of Treg in neonatal tolerance still remains unknown. Gao et al. reported that neonatal BALB/c mice injected with semiallogeneic CAF1 (BALB/c × A/J) splenocytes could induce antigen-specific tolerance to A/J mice, which lasts into adulthood. These authors demonstrated that A/J-specific anergic CD8^+^ T cells were present in neonatal primed mice that developed tolerance but not in those that rejected A/J skin grafts. Anergic CD8^+^ cells were regulated by CD4^+^CD25^+^ Treg and showed decreased proliferation and no CTL activity against A/J targets *in vitro* (18). Donor-derived Treg may be dispensable in the induction of neonatal tolerance (19), but host Treg may play an important role in the maintenance of tolerance (20).

### Th1 to Th2 Immune Deviation

**Figure 1:**
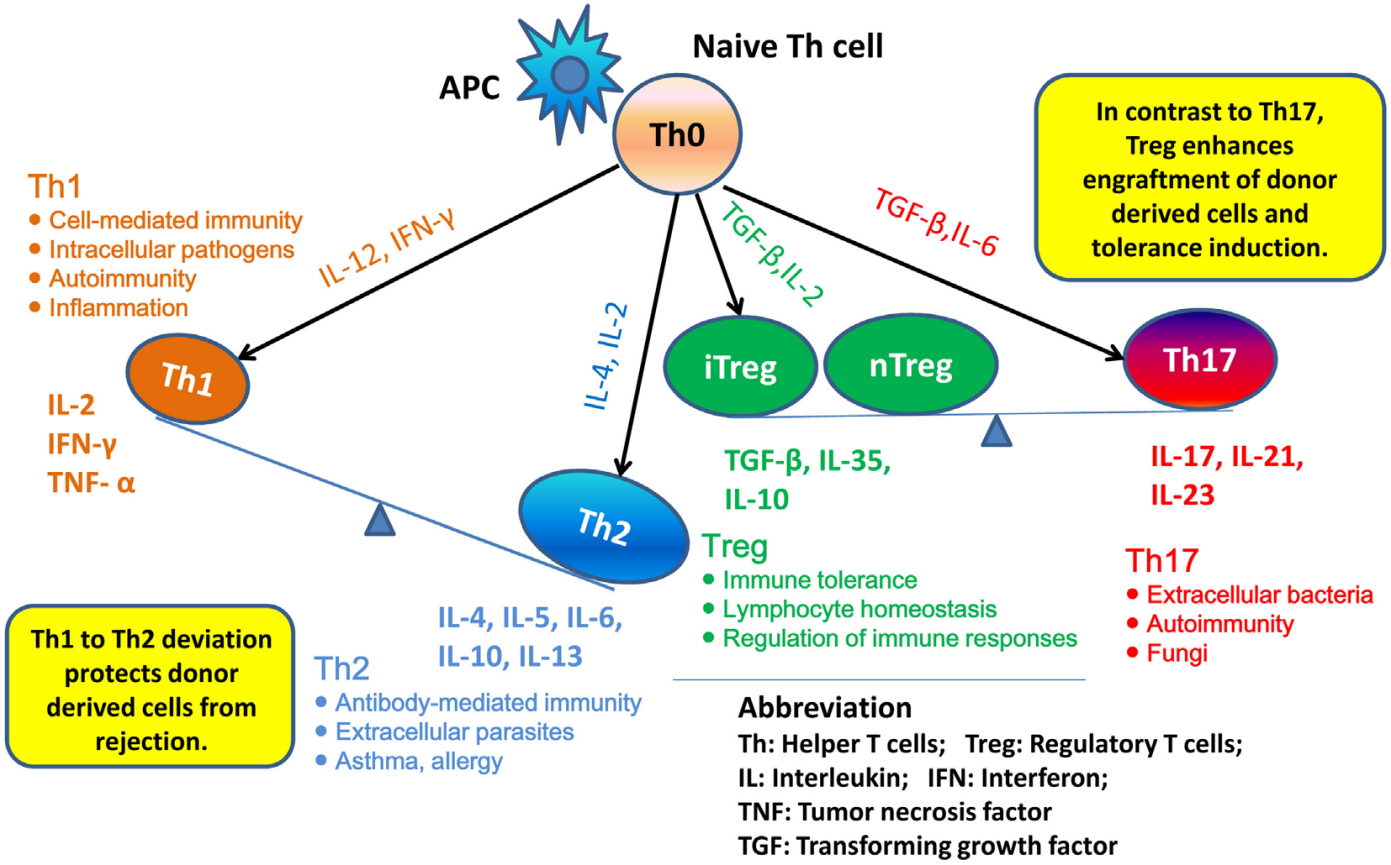
**Th1 to Th2 deviation and Th17–Treg axis**. Following activation by antigen-presenting cells (APCs), naive Th cells can be polarized into different effector T cell subsets: T helper 1 (Th1), Th2, Th17, and induced regulatory T (iTreg) cells, depending on the local cytokine environment. Besides iTreg, there is another Treg subset, “natural” Treg (nTreg), which develops as a distinct lineage in the thymus. In neonatal mice, newborn T cells generate Th2-biased immune response, thus may protect donor cells from rejection. nTreg and iTreg are critical in the mechanism of neonatal tolerance induction. They promote the donor cells engraftment and tolerance induction. By contrast, promotion of Th17 immunity can prevent establishment of lymphoid chimerism and neonatal tolerance induction.

T helper 1 to Th2 immune deviation has been proposed as another explanation for neonatal tolerance. It suggests that in neonatal mice, the newborn T cells will generate a Th2-biased immune response that will protect donor cells from rejection (Figure 1). Manifestations of Th2 cytokine bias in neonatal mice were demonstrated: (i) in the primary immune response, where CD4^+^ Th1 and CD8^+^ CTL responses are limited in neonatal mice initially inoculated with foreign antigen and (ii) in the secondary immune response, where mice immunized during the neonatal stage mount Th2-dominated memory responses when re-exposed to the same antigen in adulthood (21–23). Moreover, it was demonstrated that blocking of IL-4, the key cytokine involved in the development of Th2 cells, prevented the induction of neonatal transplantation tolerance in mice inoculated at birth with F1 splenic cells (24). In conclusion, Th1 to Th2 immune deviation at the neonatal stage is considered to have a major impact on the immediate acceptance of donor cell inoculum and the following donor-specific tolerance induction.

### Role of Th17 Cells

Besides Th1 and Th2, the role of Th17 cells in neonatal tolerance induction also appears to be important in mice. Th17 cells differentiate from naive CD4^+^ T cells and can produce IL-17, IL-21, and IL-22 (25). The cytokine IL-17 is characterized by a high inflammatory potential, as it can mobilize, recruit, and activate neutrophils that participate in allograft rejection (26). It was demonstrated in mice that, concomitantly with the development of a Th1-type response, deprivation of IL-4 upon F1 splenic cell treatment at birth resulted in the emergence of an anti-donor Th17-type response, which could be detected as early as 2 weeks of age. These observations suggest that inhibition of the neonatal Th2 response may promote Th17 differentiation and Th17 alloimmunity, which might prevent the establishment of lymphoid chimerism and neonatal tolerance induction (27). To fully address the role of Th17 in neonatal tolerance induction, we considered the Th17–Treg axis. Tregs play a critical role in maintaining self-tolerance and are now considered as an important therapeutic target to induce transplant tolerance. However, recent studies highlighted that Treg can be subverted by inflammatory conditions and converted into Th17 cells (28). Thus, a better understanding of the parameters that regulate the Th17–Treg axis may also be of interest in the exploration of the therapeutic effects of transferred Treg in transplantation (Figure 1).

### The “Danger” Model

The “danger” model highlights the role of APCs in neonatal tolerance mechanisms. Matzinger and colleagues showed that the proportion of APCs in Medawar’s typical donor inoculum (3) is the factor controlling tolerance or immune activation (29). Medawar’s splenocytes or BM cell suspensions contain a large percentage of T and B cells, but very few professional APCs (29), which cannot costimulate naive T cells (30–32). Thus, by administering a pure population of DCs, Matzinger et al. demonstrated that neonatal T cells could be activated to produce CTL responses (33). They also proposed that the number of cells administered was important, and that it was possible to induce tolerance in adults, provided that the number of “non-costimulatory cells” in the inoculum could be large enough.

When studying the “danger model,” future research may focus on the role of the innate immune system in response to transplantation. However, a consensus that has been reached is that no single theory can explain the function of the immune system as a whole. Transplant tolerance is neither a single phenomenon nor is it achieved by a unitary mechanism.

## Mixed Chimerism and Neonatal Tolerance: Implication from *In Utero* HSCT

Since it was discovered by Owen in 1945, hematopoietic mixed chimerism has been widely accepted as an important element and explanation of tolerance induction. In many experimental or clinical transplant studies, induction of mixed chimerism *via* HSCT has become the direct objective and a criterion to evaluate tolerance induction. However, the exact relationship between mixed chimerism and transplant tolerance has not been clarified. In other words, in order to induce tolerance for solid organ or composite tissue allograft, the required parameters of chimerism, such as duration (transient or durable chimerism), level (macro- or micro-chimerism), and location (chimerism in peripheral blood or chimerism in lymphoid tissue), are not completely understood. Studies on kidney transplantation in non-human primates and human patients illustrate that renal allograft tolerance can be induced in recipients even with transient chimerism (7). Nevertheless, studies on composite tissue allograft in the swine model emphasize the importance of durable chimerism to maintain the tolerance to skin components (34). This might be explained by the involvement of different tolerance induction mechanisms in the maintenance of tolerance to different allografts. Theoretically, durable chimerism may be an indicator of the acceptance of a skin graft. However, neonatal transplant tolerance protocols devoid of immunosuppressive therapy, such as those applied in rodent species, could not be effective in large animals and humans, who are born with an almost entirely functional T cell immunity, for which a durable allogeneic chimerism may indicate the necessity for a more rigorous conditioning protocol. An applicable mixed chimerism induction protocol in human neonates must balance the potential beneficial outcome and its proper harmful effects. To achieve this objective, it is necessary to evaluate the immune barrier to allogeneic cell engraftments in newborns. However, very few studies have been reported on chimerism and tolerance induction in large animals and human neonates. We thus focus on chimerism studies conducted after *in utero* HSCT (IUHSCT) in non-defective animals, in order to gain an insight into the potential outcome of HSCT in neonates.

MHC-mismatched IUHSCT in fetal mice can give rise to a high percentage of mixed chimerism detected at birth, which may decrease with age but can still maintain a long-term detectable microchimerism (35–37). Carrier et al. reported that Balb-c (H-2d) and C57BL/6 (H-2b) fetal mice, which had received intraperitoneal or transplacental transplants of either allogeneic or congenic fetal liver cells demonstrated a durable microchimerism in peripheral blood, spleen, and liver beyond 20 weeks of age. Allogeneic recipients were also evaluated for donor skin graft acceptance at 6 and 12 months of age, and authors concluded that a very small proportion of circulating donor cells in the blood or the tissues (≤0.1% donor cells) was sufficient for the induction and maintenance of tolerance. Moreover, the presence of donor cells in the circulating blood was not necessary for long-term skin graft acceptance or maintenance of permanent skin graft acceptance (35).

Studies on chimerism after xenogeneic or allogeneic IUHSCT have been reported in large animal models, including sheep (38–43), canine (44), swine (45, 46), and non-human primate (47–50) models.

Interestingly, in the study by Mathes et al., 17 outbred Yorkshire pig fetuses received at mid-gestation intravascular injections of MHC-mismatched, T cell-depleted BMCs harvested from miniature swine (46). Thirteen healthy piglets were born and nine of the newborn piglets had detectable levels of peripheral chimerism ranging from 0.16 to 1.6% at birth. However, seven piglets lost this chimerism at 6–9 weeks of age. Two animals had persistent, long-term chimerism with approximately 1% of donor cells in peripheral blood. Remarkably, in the nine piglets which were chimeric at birth, five received successful donor-matched kidney transplantation and three (with or without durable chimerism) developed donor-specific tolerance.

In non-human primates, Cowan et al. infused T cell-depleted, parental BMCs into early gestation non-defective fetal rhesus monkeys (47). Engraftment was detected in eight animals and six of them developed a durable chimerism in peripheral blood and bone marrow up to 3 years of age. Although the amount of donor cells in marrows from long-term engrafted animals was <0.1%, *in vitro* MLR and cell-mediated lymphocytotoxicity studies between the recipient and donor cells indicate that donor-specific tolerance was induced. In the study by Shields et al., allogeneic BMCs, including a T cell dose ranging from 2.6 × 10^5^ to 1.1 × 10^8^ cells/kg, were transplanted into female fetal recipients. The presence of donor T cells improved chimerism, since seven out of eight live-born neonates displayed high levels of chimerism (ranging from 0.4 to 12.5%) in cord blood or bone marrow within 1 month postpartum, although chimerism in the peripheral blood did not reach significant levels within the 2 months after birth. A lower level of alloreactivity was proven in chimeric animals compared to their donors by *in vitro* MLR. However, infusion of donor T cells also increased the risk of GvHD (49). Based on this study, Shields et al. investigated the effects of immunosuppressive therapy in fetuses for IUHSCT in non-human primates. Macaca fetuses received haploidentical, cytokine mobilized HSCT, with a combination of the corticosteroid betamethasone and rabbit anti-human thymocyte globulin. After birth, the level of chimerism in the progenitor population was higher in the immunosuppressed animals than the control animals (11.3 ± 2.7 versus 5.1 ± 1.5%) and remained significantly higher at an age of 14 months onward. This study proved the benefit of non-toxic conditioning protocols in the context of IUHSCT (50).

Studies on IUHSCT demonstrated the outcome of chimerism induction in immunocompetent fetal hosts. In large animal models, the immune barrier is more efficient at impeding the engraftment of allogeneic HSCs and at generating durable chimerism. However, in many studies, the transient microchimerism in peripheral blood or lymphoid tissues displayed a positive effect in reducing alloreactivity. In clinical studies, full acceptance of allogeneic HSCs without IS only occurs in defective fetuses or neonates with SCID (6). Thus, it can be concluded that neonatal tolerance induction protocols, devoid of conditioning therapy, are not reproducible in human neonates, which are born with an almost fully competent T cell immunity. However, studies on IUHSCT in large animal models suggest that human neonates, as immunologically immature individuals, may accept a tolerance induction protocol with non-toxic or less toxic conditioning regimens.

## Advantages of Tolerance Induction in Human Neonates

In contrast to rodent species, neonatal tolerance induction protocols were not effective in large animals or human neonates, in which/whom the immune system is more competent with regards to alloimmunity. But remarkably, compared to adult recipients, SOT or hematopoietic stem cell transplantations (HSCTs) in human neonates and infants still demonstrate several interesting phenomena, indicating that these young patients may be more susceptible to tolerance induction. For example, (i) liver transplantations in infants younger than 90 days have similar graft acceptance and patient survival compared to those in older children and in adults, although these pediatric patients have a higher chance of responding to tolerance induction and to the subsequent withdrawal of IS (51–53); (ii) ABOi organ transplantation and B cell tolerance can be achieved in human infants (5); and (iii) recipients of umbilical cord blood (UCB) cells experience less graft versus host disease (GvHD) than recipients of HSCT from an adult donor (54). Thus, an interesting issue for pediatric transplant researchers is as follows: as a high-evolved species, how much of an advantage do human neonates and young infants still possess in transplant tolerance induction? In the previous topics, the mechanisms underlying neonatal tolerance were discussed. Keeping these mechanisms in mind, the subsequent topics focus on the advantages of tolerance induction in human neonates.

### Advantage Corresponding to the Mechanism of Passive Model

In neonatal tolerance, the passive model emphasizes the defective alloimmunity of neonatal mice, but in large animals and human neonates, T cell alloimmunity is highly competent. Although human newborns have a large percentage of CD45RA^+^ naive T cells but few memory T cells, as well as a higher percentage of CD4^+^ helper T cells and lower percentage of CD8^+^ cytotoxic T cells (55–59), these T cells showed normal *in vivo* responses to allogeneic stimulators, through both T cell proliferation and cytokine production. In one of our experimental studies, newborn pigs could effectively mount acute rejection to composite tissue allograft (containing bone, muscle, and skin components) on days 4–8 after transplantation (60). However, fewer memory T cells in newborn recipients may be beneficial for transplantation (61), since T cell memory is a great obstacle to tolerance induction.

In contrast, B cell immunity is less developed and more plastic in human newborns. Neonates and very young infants are able to produce IgM, IgG, and IgA antibodies under antigen exposure. However, the neonatal humoral response often has a delayed onset, reaches lower peak levels, and has a shorter duration (58, 62, 63). The neonatal B cell pool contains a higher proportion of B1 cells, which produce natural antibodies (mainly IgM) with low-binding affinity and are not able to differentiate into memory B cells (64). The very young infants have a low level of production of natural antibodies (isohemagglutinins) against blood group antigens that are not completely expressed on their own tissues, providing them with the ability to tolerate ABOi organ transplantation (65–68).

The central tolerance mechanism is involved in the passive model, highlighting the importance of the thymus. The thymus is a relatively large organ in the newborn infant. In contrast to adult recipients, in whom the thymus has undergone a gradual involution, the existence of a potent thymus enables the very young recipients to be a special immune host during tolerance induction procedures. The thymus plays a major role in the development of self-tolerance. Thymocytes undergo both positive and negative selection, resulting in the development of a T cell repertoire with a broad range of reactivity to foreign antigens, but without reactivity to self-antigens. The thymus was proven to be critical in the induction of donor-specific tolerance (69). Proper donor antigen presentation in the thymus after HSCT or SOT has been shown to induce tolerance to allografts. Several studies proved that thymectomy could result in acute cellular rejection or failure of tolerance induction (70, 71).

### Role of Natural Treg

It has been proven that natural Treg (nTreg) may be able to facilitate allograft tolerance induction in early life stages (18, 20). Compared to induced Treg in adults, nTreg in neonates display higher levels of expression of immature markers, such as CD45RA and TCR recombination excision circles (TREC), cytotoxic T-lymphocyte antigen 4 (CTLA4), and forkhead box P3 (Foxp3). Thus, nTreg is regarded as a functionally mature population with a naive phenotype. In humans, researchers have proven that cord blood of preterm newborns contained a high proportion of CD4^+^CD25^+^ nTreg, which declined with gestational age (72). This unique population of regulatory cells may promote tolerance induction in neonates and very young infants. The thymus may also have significance in such mechanisms, since it is the source of nTreg cells (69).

### Th1 to Th2 Deviation in Human Neonates

Although widely accepted as immunocompetent, human neonatal T cell immunity is biased toward a Th2 response. Disruption of Th1 cell responses have been observed following certain infections and immunizations (73–77). But compared to mice, the Th2 bias is not as pronounced in human neonates, since lower levels of all cytokines can be detected (78). Recently, several studies have shown that neonatal B cells demonstrated an immunoregulatory function (30, 79, 80).

### Advantage Corresponding to the Mechanism of “Danger” Model

Antigen-presenting cells mainly include DCs, monocytes, and macrophages. The defective APC functions have been reviewed by Velilla et al. (81). Converse to neonatal T cells, neonatal APCs are deficient in the production of cytokines, such as TNF-α, IL-1, IL-6, or IL-12, in response to bacterial lipopolysaccharide (LPS) or CD40-signaling (75, 82–85). APCs from human cord blood also appear to be immature as they exhibit low or no basal expression of costimulatory molecules, including CD40, CD80, or CD86 (86, 87). The intrinsic deficiencies of the APC function in neonates subsequently results in the defective interaction between APCs and T cells, which could lead to secondary defects of adaptive T cell responses. Moreover, neonatal nTreg can downmodulate the function of both APCs and T cells through direct and indirect mechanisms (81).

## Tolerance Induction Approaches in Immunologically Mature Recipients

To the best of our knowledge, until now there is no study of tolerance induction through donor cells infusion conducted in pediatric patients. All of such clinical trials are performed in adult patients. Thus, we cited these clinical trials in order to discover the potential strategies for tolerance induction in infants. These clinical trials can be divided in three types: (1) tolerance induction through HSCT under non-myeloablative and lymphodepletive conditioning regimens; (2) transplantations of Treg, facilitating cells (FCs), or mesenchymal stem cells (MSCs); and (3) T cell costimulatory blockade, respectively, their principles correspond to passive model, active model, and danger model.

### HSCT *via* Non-Myeloablative and Lymphodepletive Conditioning Regimens

According to the passive model, neonatal tolerance can be induced in neonatal mice, owing to the defective immune system of the recipient. For immunologically mature recipients, tolerance induction could be achieved by mimicking such a defective immune system, by partially destroying the immune system of the recipients by a “conditioning” regimen.

#### Non-Myeloablative Conditioning Regimens in Animal Models

Following the discovery of tolerance in neonatal mice by Medawar’s team, subsequent studies revealed that tolerance can also be induced in adult mice. In 1955, Main and Prehn (88) demonstrated that under myeloablative conditioning, adult mice that received allogeneic BMC infusions were rescued from ablation and were also tolerant to donor skin grafts. Success of tolerance induction was also extended from the rodent model to large animal models. In 1972, with a canine model, Rapaport et al. confirmed that induction of unresponsiveness to canine renal allografts could be achieved by total body irradiation (TBI) and bone marrow transplantation (BMT) (89). Thereafter, induction of tolerance to organ transplants by hematopoietic chimerism was confirmed in all of the large animal models tested (90). Although myeloablative conditioning was acceptable for the use of HSCT in hematologic malignancy, the relevant risk of complications was unacceptable when applied to non-malignant situations of solid organs and composite tissue allotransplantation.

Therefore, protocols for performing HSCT for tolerance induction involving non-myeloablative conditioning became the focus of subsequent studies. Ildstad et al. first reconstituted the lethally irradiated adult mice with T cell-depleted bone marrow containing both recipient and donor components. As former study, this therapy led to long-term survival of the reconstituted animals and specific prolongation of subsequent skin grafts of donor type. Animals reconstituted in this fashion were fully reactive to third-party allografts and did not appear to manifest signs of GvHD (91). Sharabi and Sachs evaluated the outcome of tolerance induction of unmanipulated fully MHC-disparate BMT under non-lethal conditioning regimens. The results demonstrated that stable mixed chimerism with donor-specific tolerance can be induced in skin graft across an MHC barrier after a non-lethal preparative regimen, without clinical GvHD and without the risk of aplasia (92). Colson et al. also reported that durable multilineage mixed allogeneic chimerism and donor-specific transplantation tolerance for skin, and primarily, cardiac vascularized allografts can be achieved across multiple histocompatibility barriers using a non-myeloablative radiation-based regimen (93). Similarly, non-myeloablative radiation-based regimens were subsequently extended successfully to the induction of tolerance to renal transplants in fully mismatched cynomolgus monkeys (94, 95).

In the swine model, mixed chimerism across both minor and major histocompatibility barriers can be established using high doses of peripheral blood stem cells (200 × 10^8^ cells/kg) in the absence of whole body irradiation (96). Interestingly, a major finding of these studies was that animals with 1% peripheral blood donor chimerism showed just as much tolerance as those with 100% donor chimerism, suggesting that complete replacement of the recipient hematopoietic system with that of the donor is not a prerequisite to tolerance induction. It was also hypothesized that, although chimerism was required to induce tolerance, it was not necessary for its maintenance and that immune regulation would be able to maintain the tolerant state after the loss of chimerism. Donor antigen from the kidney allograft was believed to directly contribute to the regulatory mechanism (97).

#### Non-Myeloablative Conditioning Regimens in Clinical Kidney Transplantation

Tolerance induction protocols involving non-myeloablative conditioning subsequently evolved into clinical trials in the case of kidney transplantation (Table 2) after the success obtained in animal experiments. The following paragraphs focus on clinical protocols that have been performed in HLA-identical and HLA-mismatch renal transplants.

**Table 2:** **Comparison of different non-myeloablative conditioning protocols for tolerance induction in renal transplantation**.

Therapies	MGH protocol (7, 100)	Stanford protocol (98, 99)	NMH protocol (90, 103)
Renal transplantation	Related, HLA mismatched (day 0)	Related, HLA identical (day 0)	Related or unrelated, HLA mismatched (day 0)
Donor-derived cells infusion	BMCs (day 0)	G-CSF mobilized CD34 + HPCs and CD3^+^ T cells (day 0)	G-CSF mobilized, FC-based HSCs, αβ-T cells (day 1)
Lymphodepletion by mono- or poly-clonal antibodies	Rituximab (375 mg/m^2^, days −7 and −2), anti-CD2 mAb (0.6 mg/kg, days −1, 0, and 1)	ATG (1.5 mg/kg/day, days 0–5)	None
Cytoreductive medication	Cy (50 mg/kg/day, days −5 and −4)	None	Cy (50 mg/kg/day, days −3 and +3), Flu (30 mg/kg/day, days −4, −3, and −2)
Irradiation	Thymic irradiation (700 cGy, day −1)	TLI (80 or 120 cGy/day, days 1–11)	TBI (200 cGy, day −1)
Maintenance immunosuppression	Prednisone (days 0–10), CsA	Prednisone (days 0–10), CsA, and MMF	Tacrolimus and MMF

*MGH, Massachusetts General Hospital; NMH, Northwestern Memorial Hospital; HLA, human leukocyte antigens; BMC, bone marrow cell; G-CSF, granulocyte colony stimulating factor; HPC, hematopoietic progenitor cell; HSC, hematopoietic stem cell; ATG, rabbit anti-thymocyte globulin; Cy, cyclophosphamide; Flu, fludarabine; TLI, total lymphoid irradiation; TBI, total body irradiation; CsA, cyclosporine A; MMF, mycophenolate mofetil*.

In the HLA-identical renal transplantation study by Scandling et al. in Stanford University (98, 99), 16 patients conditioned with total lymphoid irradiation (TLI) and ATG were given kidney transplants and an injection of 10 × 10^6^/kg CD34^+^ hematopoietic progenitor cells and 1 × 10^6^/kg CD3^+^ T cells from HLA-matched donors. Fifteen patients developed multilineage chimerism without GvHD and eight individuals with chimerism for at least 6 months were withdrawn from anti-rejection medications for 1–3 years (mean, 28 months) without subsequent rejection episodes. Blood cells from all patients showed high ratios of CD4^+^CD25^+^ Treg and natural killer T (NKT) cells versus conventional naive CD4^+^ T cells at an early stage, and those drugs showed specific unresponsiveness to donor alloantigens. A clinical trial of HLA-mismatched renal transplantation in Massachusetts General Hospital began in 2002 (7, 100). Ten patients received combined bone marrow and kidney transplants from HLA single-haplotype mismatched living-related donors, with the use of a non-myeloablative preparative regimen similar to HLA-matched renal transplantations (Table 2). Transient chimerism and reversible capillary leak syndrome developed in all recipients. Overall, the outcome from HLA-mismatched kidney transplants is not as satisfactory as those from HLA-matched transplants: 3/10 patients experienced graft loss; 3/10 patients in the first cohort developed chronic humoral rejection and mycophenolate mofetil (MMF) was re-administrated in 2 of them; 3/10 patients in the second cohort remained stable, but a long-term monitoring was necessary; and 1/10 patient remained healthy and without rejection for over 10 years (90).

#### Lymphodepletive Conditioning Regimens

As discussed above, non-myeloablative protocols for tolerance induction were introduced in experimental BMT studies. However, conventionally, conditioning of the recipient still required TBI, thymic irradiation, or cytotoxic drugs, such as cyclophosphamide. The potential toxicity associated with cytoreductive conditioning is a major reason preventing these protocols from being routinely implemented in the clinical context. To increase the potential clinical acceptability of such regimens, it would be desirable to improve the success rate of such protocols and to achieve chimerism and tolerance avoiding cytoreductive conditioning.

Conception of lymphodepletion-based conditioning regimen was developed under such circumstances. In 1970, Gozzo and Monaco reported that allogeneic, homozygous donor BMC infusion under lymphocyte-depleting condition with antilymphocyte serum (ALS) significantly prolonged graft survival time in mice (101). Later, Monaco et al. introduced similar conditioning and BMT protocols to human kidney transplantation (102). One recipient was given ALS (first 14 days after renal transplant) and 11 × 10^9^ donor BMCs (at day 25 post-operation) along with conventional doses of prednisone and azathioprin. The conventional immunosuppressive agents were tapered, and renal function was normal 8 months following transplantation. Histological examination of the renal allograft showed only minimal evidence of rejection.

Nowadays, this lymphodepletion-based conditioning regimen has been successfully implemented in HLA-identical renal transplantation in the clinical trial of the Northwestern University, which was initiated in 2008 (90, 103). In this study, 20 cases of HLA-identical sibling donor–recipient pair renal transplants were performed using this conditioning regimen. Lymphodepletion was induced by administering alemtuzumab, and four infusions of donor CD34-selected HSCs were then performed postoperatively during the first 9 months following renal transplantation. Conventional IS was tapered and converted to sirolimus monotherapy. Transient microchimerism was the only phenomenon present, and it had disappeared at the 1-year follow-up examination. IS is generally withdrawn within 24 months in subjects with stable renal function and normal protocol biopsies. At the time of this publication, patients in the study ranged from 2 to 52 months posttransplant and 50% of these recipients have taken off immunosuppressive agents for over 1 year. None of the 20 subjects displayed any deterioration of their renal function, such as that occurring in the early posttransplant period. Increased numbers of CD4^+^CD25^high^FoxP3^+^ Treg were observed in the peripheral blood of these patients during lymphoid reconstitution, suggesting the induction of an immunoregulatory state. This tolerance induction protocol excludes the need for permanent chimerism but focus on a prolonged immunoregulatory environment, which could be provided by donor HSC infusions in the setting of HLA-identical renal transplantation.

### Transplantations of Treg, FCs, or MSCs for Tolerance Induction

The active model of neonatal tolerance that focused on the function of “suppressor cells” has been proven to be wrong. However, the concept of “suppressor cells” continues to develop and has resulted in the discovery of Treg. If we extend the notion of active model, which emphasizes the tolerogenic cells in the inoculum, we can include several cell therapies, namely, transplantation of Tregs, FCs, and MSCs. These cell therapies have been introduced into clinical trials for the purpose of tolerance induction in BMT, SOT, or autoimmune diseases.

#### Transplantation of Treg

Regulatory T cells have been shown to be efficient in controlling auto- and alloimmunity in preclinical studies and have recently been included in clinical studies. To date, approximately four clinical trials have been reported using Treg therapy in humans, three in GvHD (104–106) and one in type 1 diabetes (107). Clinical trials involving the infusion of Treg for promoting tolerance induction to solid organ allograft have not been reported but have been registered in www.clinicaltrials.org, including liver transplantation (NCT 01624077) and kidney transplantation (NCT 01446484).

A clinical trial of *ex vivo* expanded Treg was initially reported by Trzonkowski et al. in 2009, for the treatment of GvHD (104). In this study, two patients who suffered from either acute or chronic GvHD were enrolled. CD4^+^CD25^+^CD127^−^ Tregs were sorted from buffy coats taken from two family donors, expanded *ex vivo*, and transferred to respective recipients. This therapy significantly alleviated the symptoms and reduced pharmacologic IS in the case of chronic GvHD, while in the case of grade IV acute GvHD it only transiently improved the condition. Brunstein et al. first evaluated the safety profile and therapeutic effects of UCB Treg in 23 patients (105). CD4^+^CD25^+^FoxP3^+^ Tregs from cryopreserved UCB were *ex vivo* expanded and then infused into recipients after double UCB transplantation. Following Treg infusion, there was a reduced incidence of grade II–IV acute GvHD without any deleterious effects on the risk of infection, relapse, or early mortality. In the trials of Di Ianni et al., the impact of donor Treg was studied in the context of HLA-haploidentical SCT (106). Twenty-eight patients received infusion of immunoselected CD4^+^CD25^+^ Treg, followed by transplantation of immunoselected CD34^+^ cells and mature CD4^+^CD25^−^ T cells from the same donor. Results showed that (i) 26/28 patients were engrafted, (ii) acute GvHD developed in only 2/26, and (iii) no patient developed chronic GvHD. In addition, in the trials of Marek-Trzonkowska et al., autologous CD3^+^CD4^+^CD25^high^CD127^−^ Tregs were sorted from peripheral blood and used to treat type 1 diabetes in children, resulting in a highly satisfactory outcome (107).

Interestingly, recent researches showed that chimeric antigen receptors (CARs) may be an effective way to produce a sufficient amount of antigen-specific Tregs for clinical applications. CARs are created by combining antibody variable domains with TCR-signaling domains (108, 109). Furthermore, with their high level of flexibility but specific targeting, clustered regularly interspaced short palindromic repeats (CRISPRs) and CRISPR-associated systems (Cas), which have become powerful tools for genome editing, might play an important role in the production of engineered antigen-specific Tregs (110).

A major question for Treg therapy is that Tregs can be converted into Th17 cells under inflammatory conditions. Therefore, it is, important to study whether the adoptively transferred Treg lines can be diverted into a Th17 response and fulfill their role in the response to organ transplantation. The development of therapeutic strategies focusing on the shift of the Th17–Treg axis toward the stabilization of the Treg population would be an interesting future prospective.

#### Co-Infusion of FCs in HSCT

CD8^+^/TCR^−^ FCs have been shown, in experimental studies, to potently enhance engraftment of allogeneic HSCs without causing GvHD (111, 112). The predominant subpopulation of FCs resembles plasmacytoid precursor dendritic cells (pDCs) (113). Bone marrow-derived plasmacytoid dendritic cells induce naive T cells to differentiate to become antigen-specific Treg, thus facilitating the induction of allogeneic HSCs engraftment (114). Co-infusion of FCs may circumvent major issues raised by tolerance induction of solid organ or composite tissue allograft through HSCT, by improving chimerism, and concomitantly by using less toxic conditioning regimens. Recently, clinical trials based on FC infusions with HSCT have been conducted, and the outcome of this trial proves this may be a promising therapy for tolerance induction in pediatric recipients (115, 116).

A novel approach was reported by Leventhal et al. who bioengineered human marrow to remove GvHD-inducing immune cells and to preserve FC, HSC, and progenitor cells. This approach was followed by the fist clinical trial based on myeloablatively conditioned, mismatched BMT in 54 recipients with hematological malignancies. Results confirmed the function of FCs in facilitating engraftment and avoiding the occurrence of GvHD (90). The success of this first resulted in the implementation of subsequent clinical trials using FCs for tolerance induction in kidney transplant recipients (116). Fifteen HLA-mismatched, -related, and -unrelated subjects were enrolled. Conditioning consisted of three doses of fludarabine on days −4, −3, and −2; two doses of cyclophosphamide on days −3 and +3; and 200 cGy TBI on day −1. Administration of tacrolimus and MMF was initiated on day −3 and was continued in order to maintain IS after the transplant procedure. Kidney transplantation was performed (day 0) without antibody induction or oral corticosteroid therapy. The bioengineered HSC product, enriched for FCs, was infused intravenously on the day after kidney transplantation. The conditioning was well tolerated with outpatient management after day 2. In follow-up examinations, high levels of peripheral blood chimerism (6–100%) were observed in 14/15 patients. All subjects demonstrated donor-specific hyporesponsiveness and were weaned from full-dose IS. Complete withdrawal of IS was successfully accomplished 1 year after transplantation in all of the subjects with durable chimerism. There has been no occurrence of GvHD or engraftment syndrome. These results suggest that manipulation of a mobilized stem cell graft and non-myeloablative conditioning represent a safe, practical, and reproducible means of inducing durable chimerism and donor-specific tolerance in SOT recipients.

#### Transplantation of MSCs

Findings obtained from experimental studies of SOT or HSCT proved that MSCs facilitate the induction of transplant tolerance (117). Clinical studies of MSCs transplantation in SOT or HSCT recipients are currently underway, and preliminary results from the clinical trials have been reported (118).

In the clinical trial of Perico et al., autologous MSC infusion was conducted in two kidney transplant recipients (119). Patients were given T cell-depleting induction therapy, and IS was maintained using cyclosporine A (CsA) and MMF. On day 7 posttransplant, MSCs were administered intravenously. Findings from this study show that MSC infusion in kidney transplant recipients is feasible; it allows enlargement of Treg in the peripheral blood and controls the function of memory CD8^+^ T cells. However, serum creatinine levels increased 7–14 days after cell infusion in both MSC-treated patients. A graft biopsy in one patient excluded acute graft rejection but showed a granulocyte-mediated focal inflammatory infiltrate. Of note, an experimental study conducted on murine kidney transplant showed that a single administration of MSC before (day 1), but not after renal transplantation, avoided the acute deterioration of graft function while maintaining the immunomodulatory effect associated with MSC treatment (120). In subsequent trials, two kidney transplant recipients were given pretransplant (day 1) infusion of BM-derived autologous MSC before T cell-depleting induction therapy (121). In the first patient, MSC treatment was uneventful and graft function was normal during the 1-year follow-up. In the second patient, however, acute cellular rejection occurred 2 weeks after posttransplant. These studies indicate that autologous MSC may have a low capacity to control the host immune response at an early-stage posttransplantation in the context of a high alloreactive environment.

A clinical trial conducted by Tan et al. showed that during the 1-year follow-up, MSC-treated patients had a significantly lower risk of opportunistic infections than those not receiving the MSCs infusion (122). However, in the study by Reinders et al., six renal transplant recipients who were given two doses of autologous BM-MSCs showed signs of subclinical rejection and/or an increase in interstitial fibrosis/tubular atrophy in renal biopsies, 4 weeks or 6 months posttransplantation. Besides, five out of six patients displayed a donor-specific downregulation in the *ex vivo* peripheral blood mononuclear cell (PBMC) proliferation assay, whereas three patients developed an opportunistic viral infection (123). Authors concluded that MSC could induce over-IS. These studies suggest a careful monitoring of the side effects of MSC therapies, especially in chronically immunosuppressed transplant recipients, who are already at an increased risk of contracting infections and malignancies (118, 124).

### T Cell Costimulatory Blockade in Organ Transplantation

According to the “danger model” theory, T cell costimulatory signals play an important role in neonatal tolerance induction (33). Theoretically, donor BMT combined with the administration of T cell costimulatory blockade agents could thus, at least partially, mimic the conditions in neonatal mice, facilitating tolerance induction in the immunologically mature recipients. Currently, tolerance induction protocols with non-lymphodepletive, non-toxic conditioning regimens have been developed, mainly containing costimulation blockade agents and routine immunosuppressive drugs.

In the mouse model, Blaha et al. introduced a tolerance induction protocol, including BMC infusions, anti-CD40L monoclonal antibody (blockade of CD40: CD154 costimulatory pathway), and CTL-associated protein 4 (CTLA4)-Ig (blockade of CD28: B7 costimulatory pathway) administration in addition to short-term IS (rapamycin, methylprednisolone, and MMF for 4 weeks after BMT). This protocol led to long-term multilineage chimerism in 28/30 recipients and to the significant prolongation of skin graft survival (125).

In a large animal model, Wachtman et al. confirmed the beneficial effect of CTLA4-Ig for inducing tolerance to vascularized composite allografts (VCA) in swines (126). In this study, miniature swines received MHC-mismatched hind limb VCA and were administered with CTLA4-Ig on days 0, 2, 4, and 6, in addition to a 30-day treatment course of tacrolimus, though no cytoreductive conditioning regimen or extra donor BMCs infusions were given. The recipients demonstrated indefinite prolonged muscle survival, and the skin component also displayed remarkably prolonged survival. Although further study on the underlying mechanisms is required, a possible explanation might be that the bone marrow component in the hind limb allograft might have benefited from the costimulatory blockade protocol and, therefore, microchimerism was developed to regulate the recipient alloimmune response.

In non-human primates, Lo et al. developed a belatacept-based regimen without HSCT in rhesus monkeys. The rhesus monkeys studied, received MHC-mismatched renal allotransplants with IS containing belatacept, mammalian target of rapamycin inhibitor, and sirolimus. This therapy successfully prevented allorejection in all animals. However, tolerance was not induced, since allografts were rejected after withdrawal of IS, indicating the important role of HSC infusions (127). In a MHC-defined rhesus macaque BMT model, Page et al. evaluated a non-lymphodepletive conditioning protocol combined with the administration of the CD40 monoclonal antibody, CTLA4-Ig, and sirolimus to produce mixed chimerism by HSCT. Results showed that prolonged HSC engraftment required the presence of all three agents during maintenance therapy and resulted in graft acceptance for the duration of immunosuppressive treatment. In this study, although complete withdrawal of IS was not achieved, notably, this protocol excluded the use of calcineurin inhibitors (CNIs) and steroids (128).

Recently, CD28 blockade has been successfully transposed to the clinical setting with the commercialization of belatacept. Transposition of blockade of the CD154:CD40 pathway has been less successful, due, in large part, to thromboembolic complications associated with anti-CD154 antibodies. Transposition of CD40 blockade has also been slow, partly due to the fact that the synergy between CD40 and CD28 blockade had not yet been demonstrated in either primate models or humans. So far, tolerance induction protocols based on costimulatory blockade agents have not yet reached the stage of clinical trials. Remarkably, however, in maintenance therapy, conversion from CNI-based IS to belatacept has been performed in renal transplant recipients, which may significantly reduce the side effects of chronic CNI therapies, such as hypertension, new-onset diabetes, tremor, and thrombotic microangiopathy, and can thus improve long-term allograft function and patient health (129).

## Experience and Strategy of Pediatric Tolerance Induction

Current improvements in immunosuppressive and antiviral regimens, advances in surgical or organ preservation techniques, and progress in donor organ allocation have significantly prolonged the survival times of both the patient and the allografts (130). Thus, children and adolescent transplant recipients are confronted with increased risks of opportunistic infections and tumors, aggravated long-term side effects and toxic effects of immunosuppressive medications, including impact on growth. They are also challenged by graft vasculopathy and chronic rejection. Besides, adolescents have the worst patient and graft survival, mainly as a result of non-adherence. Taken together, young pediatric recipients have a greater demand for successful transplant tolerance protocols (131).

To date, clinical trials of tolerance induction through HSCT are mostly conducted in adult patients in Massachusetts General Hospital (7, 100), Stanford University (98, 99, 132), and Northwestern Memorial Hospital (116), as previously mentioned. However, a safe and effective tolerance induction protocol in pediatric patients has not been defined. In pediatric SOT, only a few studies regarding tolerance induction or withdrawal of IS have been reported, most of which are concerning to liver transplantation, because of the immune-privileged status of the liver (131, 133–136).

In pediatric living donor liver transplantation (LDLT), the success rate of IS withdrawal (operational tolerance) is about 20%, which is thus higher than adult recipients (131). Although no tolerance induction regimen was administered, HSCs within the transplanted liver could have contributed to the development of chimerism and to this high “tolerance rate.” Typically, Alexander et al. reported that a 9-year-old girl with acute fulminant hepatitis after a non-specific viral illness developed complete hematopoietic chimerism and tolerance after receiving a liver allograft from a deceased male donor, with no evidence of GvHD (134). Two cases of pediatric liver transplant combined with HSC infusions have been reported and both of them achieved functional tolerance and showed an excellent clinical outcome (133, 135). In the report of Matthes-Martin et al., a 4-month-old girl with familial hemophagocytic lymphohistiocytosis received HLA-mismatched, maternal LDLT. After transplantation, the presence of maternal cells in the peripheral blood was observed following microchimerism analysis. The patient then received SCT from the same donor. Conditioning regimen consisted of busulfan, cyclophosphamide, thiothepa, and ATG. Four months after SCT, the patient was disease-free, with complete donor chimerism in bone marrow and stable hepatic graft function without any immunosuppressive therapy (135).

When other SOTs are considered, clinical operational tolerance is significantly less common. After the first successful pediatric renal transplantation was performed in 1954, only sporadic cases of operational tolerance to renal allograft have been documented, as review by Orlando et al. in 2008 (136). In a report by Roussey-Kesler et al., two recipients received deceased donor kidney transplant at pediatric age. In one patient, IS was interrupted because of non-compliance and had a stable graft function 16 years after withdrawal. The other patient developed posttransplantation lymphoproliferative disorder (PTLD) 8 years after transplant, relative to Epstein–Barr virus infection. At the time of the report, he was 8 years after PTLD treatment and he had stable renal function (137). However, studies of IS minimization have achieved great success in renal transplant recipients, including the pediatric population (138). Steroid-withdrawal (139) or steroid-free IS (140) protocol, CNI-free IS protocol (completed clinical trial NCT00023231 on www.clinicaltrials.gov) have been studied for pediatric renal transplantation.

Besides, clinical operational tolerance has never been reported in intestinal, islet, or whole organ pancreas transplantation, whereas two exceptional cases of IS withdrawal have been described after lung (141) and heart transplantation (142).

## Thymic Manipulations as an Approach for Tolerance Induction in Infants

The thymus is a highly developed lymphoid organ in the neonatal stage, making infants the ideal recipients for organ transplantation. The existence of a functional thymus may also improve the outcome of central tolerance induction by intrathymic depletion of alloreative T cells, as it has frequently been proven that the thymus plays an indispensable role in preclinical studies of donor-specific tolerance induction. According to previous experimental and clinical studies aimed at exploring central tolerance in humans, two main manipulations involving in the thymus have been reported, namely, donor thymic tissue transplantation and intrathymic injection of donor antigen.

### Donor Thymic Tissue Transplantation

Thymic tissue transplantation has been studied since the 1960s, when prior to the success of the BMT, many attempts were made in order to reconstitute the immunological capacity in infants with lymphopenic immunological deficiency by implanting fetal or infantile tissues and/or adult blood or bone marrow (143). In 1969, De Koning reported a case of transplantation of fetal thymus and bone marrow cells in a 5-month-old infant with lymphopenic immunological deficiency, and the clinical outcome was excellent (144). However, the thymus transplantation procedures were performed in the scope of BMT and not aimed at donor-specific tolerance induction.

Hence, early thymus transplantations were conducted in mice. So far, inducing donor-specific tolerance by thymic tissue transplantation across allogeneic and xenogeneic barriers has been achieved in immunocompetent mice after thymectomy, followed by lethal whole body irradiation (145, 146), TLI (147), or T cell and NK cell depletion with monoclonal antibodies (148). Non-vascularized thymic tissue allograft was subsequently extended to large animal models (149–151). Typically, Haller et al. reported thymic transplantation across an MHC class I barrier in miniature swine and concluded that thymic transplantation could serve as part of a regimen to induce donor-specific tolerance to xenogeneic organ grafts (149). In the context of heart transplantation, Johnston et al. developed a novel technique, in which the donor heart and en-bloc thymus grafts were prepared allowing the preservation of the entire arterial supply and venous drainage of the right thymic lobe (152). This technique thus enabled donor thymus transplantations that may prove to be useful in human heart transplantation.

Donor thymus co-transplantation with solid organ for tolerance induction, although shown to be effective in small and large animal models, has so far never been performed in the context of clinical organ transplantation. Currently, thymus transplantation only serves as a treatment for pediatric patients with profound primary immune deficiency due to primary athymia and the resulting lack of functional T cells, such as DiGeorge syndrome. The main complication that should be addressed is GvHD (153).

### Intrathymic Injection of Donor Antigen

An earlier study focusing on central tolerance induction by intrathymic injection of donor antigen was reported by Remuzzi et al. (154). In this study, isolated glomeruli from Brown-Norway (BN) rat kidney were inoculated into the thymus of MHC-mismatched Lewis rats pretreated with CsA for 2 days and administered subcutaneous dexamethasone at the time of inoculation. Ten days later, the kidneys of the BN rats were transplanted into the intrathymic injected Lewis rats. Donor-specific unresponsiveness allowed the renal allograft to survive indefinitely without further IS. Many other studies in rodent models have also shown that intrathymic injection of donor antigen in various forms, such as donor spleen cells, dendritic cells, or MHC allopeptides, could induce donor-specific tolerance to organ allografts (155–158).

Although intrathymic injection of donor antigen has been demonstrated to be very effective in rodents, successful studies in large animals have not been reported. But interestingly, intrathymic injection has been applied to humans in a clinical trial of pediatric heart transplantation reported by Remuzzi et al. (159). Thirty-seven children (median age of 7.4 years) entered the study. Fourteen patients received intrathymic inoculation of 8 × 10^7^ cells/kg of unmodified donor BMCs prior to sternal closure; 23 patients for whom marrow could not be harvested acted as controls. All patients received standard tacrolimus-based IS without induction therapy. Freedom from acute cellular rejection and the number of rejection episodes was not different between the study group and control group in the first 6 months after transplant. However, there was a greater freedom from late acute cellular rejection (beyond 1 year) in the study group. Among the 13 survivors in the study group, only 2 episodes of late acute cellular rejection were reported. By contrast, 20 episodes were detected in the 22 surviving controls. However, serial MLR using irradiated donor and third-party splenocytes showed no evidence of increased hyporesponsiveness in the intrathymic injection group. This clinical trial indicated that intrathymic injection with donor bone marrow is feasible and safe in the setting of pediatric heart transplantation. However, the outcome was not promising enough to allow this approach to be widely performed with other organ transplantations, such as renal transplant. Indeed, if we consider that except for heart transplant, organ transplantations do not share the same surgical access with thymic injection and intrathymic injection, these interventions would thus be either too invasive in the case of open-chest surgery or almost impossible to achieve right orientation to thymus. Several other disadvantages may include the fact that (i) intrathymic injection cannot be performed in adult patients in whom the thymus has undergone involution and (ii) intrathymic injection can neither eliminate the donor-reactive T-lymphocytes that preexisted nor prevent their expansion into the peripheral blood.

## Conclusion

The first pediatric SOT was performed in Paris in 1953 by Michon and Hamburger et al., but this mother-to-son living kidney transplant failed because of the lack of immunosuppressive medication (160). Since then, enormous progress has been accomplished in the domain of pediatric transplantation. Based on the infusion of donor hematopoietic cells and mixed chimerism creation, several tolerance induction approaches have been successfully implemented in adult renal transplantation, although accompanied with certain complications, such as engraftment syndrome. However, so far, no tolerance induction protocol has been tested for pediatric SOT.

Young pediatric transplant recipients have a great demand for tolerance induction regimens. They required longer survival time of allograft and may thus suffer more undesirable effects and risks related to long-term IS. Furthermore, they are more susceptible to chronic rejection, allograft amputation, and re-transplantation. However, newborn infants possess more advantage for central tolerance induction, because of their premature immune system and their highly developed thymus. Approaches of tolerance induction *via* hematopoietic cell therapies have achieved promising results in renal transplants in adults, and they should be evaluated in young pediatric recipients, if less toxic but effective conditioning regimens could be defined for these vulnerable children.

However, we must realize that the mechanisms underlying immune tolerance may vary between different solid organ allotransplants or composite tissue allotransplants. We may not be able to directly extend the experiences gained from tolerance induction in renal allografts to other types of allografts but have to investigate more specific tolerance induction protocols and IS withdrawal programs. Besides, for such applications in human infants, we must be able to recognize the tolerance-vulnerable patients and at least at the beginning, try to find better HLA-matched donors. In this regard, some criteria and potential biomarkers have been identified (131, 161). However, the identification of suitable biomarkers for the minimization or weaning of IS remains a challenge, which should be one of the main focuses of future studies.

## Author Contributions

HP and M-CM: substantial contributions to the conception and design, acquisition of data, analysis, and interpretation of data; drafting the article or revising it critically for important intellectual content; and final approval of the version to be published. AG, J-MD, AB, and MM: drafting the article or revising it critically for important intellectual content and final approval of the version to be published.

## Conflict of Interest Statement

The authors declare that the research was conducted in the absence of any commercial or financial relationships that could be construed as a potential conflict of interest.

## Abbreviations

ABOi, ABO-incompatible; ALS, antilymphocyte serum; APCs, antigen-presenting cells; BMCs, bone marrow cells; BMT, bone marrow transplantation; BN, Brown-Norway; CARs, chimeric antigen receptors; Cas, CRISPR-associated systems; CNIs, calcineurin inhibitors; CRISPRs, clustered regularly interspaced short palindromic repeats; CsA, cyclosporine A; CTL, cytotoxic T lymphocyte; CTLA4, cytotoxic T-lymphocyte-associated protein 4; DCs, dendritic cells; FCs, facilitating cells; GvHD, graft versus host disease; HSC, hematopoietic stem cell; HSCT, hematopoietic stem cell transplantation; IS, immunosuppression; IUHSCT, *in utero* hematopoietic stem cell transplantation; LDLT, living-donor liver transplantation; MLR, mixed lymphocyte reaction; MMF, mycophenolate mofetil; MSCs, mesenchymal stem cells; mTECs, medullary thymic epithelial cells; NKT, natural killer T; PBMCs, peripheral blood mononuclear cells; pDCs, precursor dendritic cells; PerC, peritoneal cavity; PTLD, posttransplantation lymphoproliferative disorder; RBCs, red blood cells; SCID, severe combined immunodeficiency; SCT, stem cell transplantation; SOT, solid organ transplantation; TBI, total body irradiation; TCR, T cell receptor; Th1 cell, T helper 1 cell; TLI, total lymphoid irradiation; Tregs, regulatory T cells; UCB, umbilical cord blood; VCA, vascularized composite allografts.
